# Mantle Cell Lymphoma Presenting as Diarrhea in a Liver Transplant Recipient

**DOI:** 10.14309/crj.0000000000000635

**Published:** 2021-07-21

**Authors:** Abdelwahab Ahmed, Abdullah Naji, Jinyu Zhang, Mohammad Raoufi, Mohamed Alhamar, Reena Salgia, Keith Mullins

**Affiliations:** 1 Department of Medicine, Northwestern University Feinberg School of Medicine, Chicago, IL; 2 Anesthesiology and Perioperative Medicine, Oregon Health Science University Hospital, Portland, OR; 3 Division of Gastroenterology and Hepatology, Henry Ford Hospital, Detroit, MI; 4 Division of Pathology, Henry Ford Hospital, Detroit, MI

## Abstract

We present a 63-year-old man with a medical history of hepatocellular carcinoma who underwent orthotopic liver transplant 10 years prior on long-term immunosuppressive therapy. The patient presented to the clinic with diarrhea, and the workup revealed mantle cell lymphoma. Mantle cell lymphoma is an extremely rare finding in transplanted livers. It is essential to include mantle cell lymphoma, along with a broad differential, during the workup of diarrhea in post-transplant patients.

## INTRODUCTION

Solid organ transplants are life-saving surgeries that involve a complex interplay between the doctor, patient, and donor. Diarrhea frequently occurs after solid organ transplants.^1^ One retrospective study has shown that diarrhea is the most common gastrointestinal complication in liver transplant recipients, with infections and post-transplant medications being the leading causes.^1,2^ Rare causes of diarrhea in liver transplant recipients include lymphoproliferative diseases, such as mantle cell lymphoma (MCL).^3^ MCL is a subtype of non-Hodgkin lymphoma (NHL), which develops because of abnormalities involving B cells.^4^ MCL tends to occur in patients in their 60s, is predominant in men at a ratio of 2:1, and presents with B symptoms, such as fever and fatigue.^5^ Because of the long-term use of immunosuppressive medications to prevent rejection, the risk of NHL in solid organ transplant recipients increases by 6 times relative to the general population.^6,7^ However, in contrast to various subtypes of NHLs, the incidence of MCL in solid organ transplant recipients does not significantly increase.^6^ Hence, we present a rare case describing a 63-year-old man who received a liver transplant and was found to have diffuse MCL after presenting with chronic diarrhea.

## CASE REPORT

A 63-year-old orthotopic liver transplant patient with a medical history of hepatitis C cirrhosis complicated by pretransplant hepatocellular carcinoma presented to the clinic with chronic nonbloody diarrhea. His pretransplantation surgical history included a right hepatic lobe segmentectomy and distal pancreatectomy for an isolated islet cell tumor. Two years after this surgery, radiologic evaluation revealed evidence of growing cancer in the liver. Initial therapy for his hepatocellular carcinoma was chemoembolization. The patient subsequently received a liver transplantation in 2010 with thymoglobulin induction. Both the donor and the recipient had negative serologic testing for hepatitis C virus, Epstein-Barr virus, and cytomegalovirus.

Three months before initial presentation with diarrhea, abdominal magnetic resonance imaging was completed for chronic mild elevations in liver function tests and revealed stable prominent mesenteric lymph nodes, the largest being 11 mm, of unclear etiology. His presenting symptoms included more than 6 loose stools a day, bloating, cramping more than 8 months, and weight loss of 15 pounds. On presentation, the patient's aspartate aminotransferase level was 37 IU/L, and his alanine transaminase level was 75 IU/L. The patient reported no other B symptoms. There were no concerning findings on abdominal examination. At the time of presentation, his immunosuppressive regimen consisted of mycophenolate mofetil 500 mg twice daily, cyclosporine 100 mg twice daily, and ursodiol 300 mg twice daily. The patient endorsed taking only half of the prescribed dosage because of a miscommunication. No stool studies were performed because the patient's chronic diarrhea was thought to be due to causes other than infection. However, bulking agents were prescribed, which improved the patient's diarrhea, and endoscopic evaluation was scheduled for 1 month later.

**Figure 1.**
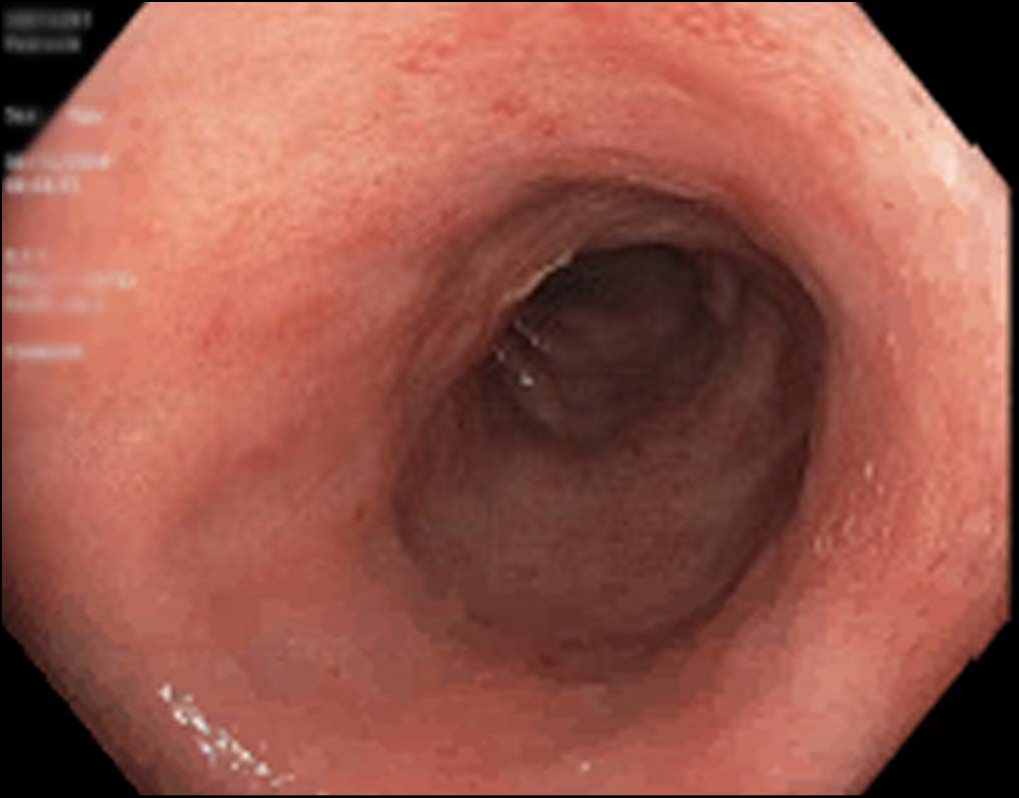
Image of the sigmoid colon revealing the areas of erythematous mucous.

**Figure 2.**
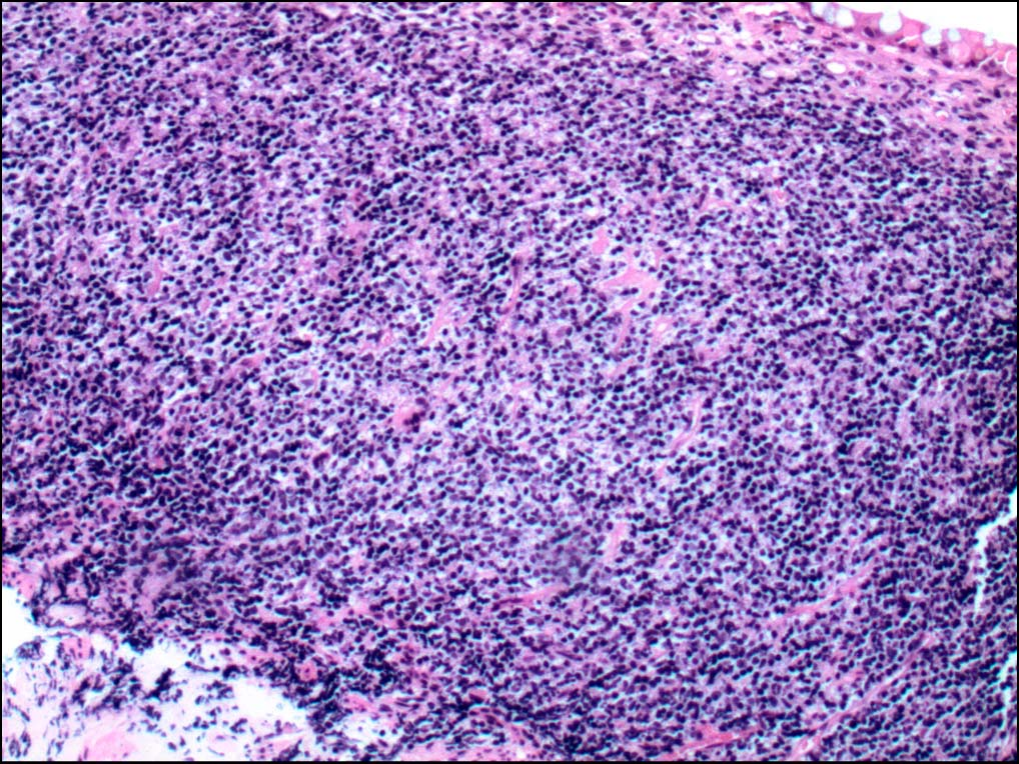
Colonic mucosa with a prominent infiltrate of small atypical lymphocytes that have round to irregular nuclei, mature chromatin, and scant to moderate clear cytoplasm (hematoxylin and eosin stain, 10× magnification).

Endoscopic evaluation revealed duodenal inflammation and mild continuous erythema in the rectosigmoid colon up to 30 cm from the anal verge (Figure 1). Biopsies of the duodenum, right colon, and left colon were performed. Histologic sections of colonic mucosa revealed prominent infiltrate of small atypical lymphocytes with round to irregular nuclei, mature chromatin, and scant to moderate clear cytoplasm (Figure 2). There was no evidence of intraepithelial lymphocytes and villous atrophy to suggest celiac disease. Immunostaining of the biopsies was negative for cytomegalovirus.

The biopsy results were indicative of lymphoma, which prompted a bone marrow biopsy. Pathology revealed that 10% of the marrow was mantle cell; fluorescence in situ hybridization revealed an 11;14 chromosomal translocation. The patient was diagnosed with stage IV MCL with diffuse gastrointestinal involvement; all biopsies from the endoscopic evaluation were retrospectively examined and tested positive for MCL. Treatment was then tailored to the patient's new diagnosis and included ibrutinib (140 mg daily) to target MCL and an antirejection regimen of cyclosporine (150 mg twice daily) and prednisone (60 mg daily). The patient's most recent bone marrow biopsy revealed residual disease, and the current plan is to have the hematology team follow the patient clinically.

## DISCUSSION

Patients who develop post-transplant MCL may present with B symptoms, such as diarrhea, gastrointestinal bleeding, and hypoalbuminemia.^8^ Although our patient was within the typical age range for developing MCL, his presentation of diarrhea 10 years after transplantation in the absence of many classic B symptoms made the diagnosis of MCL clinically challenging. The typical differential diagnosis for post-transplant diarrheal disease is an opportunistic infection from pathogens, such as *Clostridium difficile* and cytomegalovirus, graft-vs-host disease, mycophenolate-induced injury, and microscopic colitis.^9^ Therefore, MCL within the context of diarrheal disease post-transplantation has a high likelihood of being overlooked within the initial differential diagnosis.

On the suspicion of post-transplant lymphoproliferative disease, upper and lower endoscopies with biopsies are advised because they can highlight noninfectious causes, including MCL.^10^ Infection with Epstein-Barr virus also substantially increases the suspicion of lymphoid malignancies,^9^ and notably, neither our patient nor his transplant donor was positive for this pathogen. The molecular hallmark of MCL is an 11;14 chromosomal translocation,^11^ which was observed in our patient; thus, genetic testing can be informative. Hence, many lines of inquiry can assist in the diagnosis of post-transplant MCL.

Treatment strategies for MCL in liver transplant recipients involve adjusting immunosuppressive medications and initiating chemotherapy. Reduction of immunosuppressive therapy may be considered; however, because MCL is not a typical manifestation of post-transplant lymphoproliferative disease, this treatment modality should be approached with caution because targeted approaches are available.^12^ A study of 111 patients with MCL showed that those taking ibrutinib had an overall response rate of 68% and a complete response rate of 21%.^13^ Although ibrutinib has shown promising results in patients with MCL, incidents of severe hepatotoxic reactions have occurred.^14^ Thus far, our patient has not had any drug-induced liver injury from ibrutinib therapy.

In conclusion, we have reported a rare case of diarrhea secondary to MCL in a 63-year-old male orthotopic liver transplant recipient in the setting of long-term immunosuppressive medication. Our patient's case has added to the limited but growing knowledge of MCL in liver transplant recipients who present with diarrhea.

## DISCLOSURES

Author contributions: A. Ahmed and A. Naji wrote the article. J. Zhang, R. Salgia, and K. Mullins edited the article. M. Raoufi and M. Alhamar provided the images. K. Mullins is the article guarantor.

Acknowledgment: We would like to acknowledge Karla Passalacqua, PhD, at Henry Ford Hospital for editorial assistance.

Financial disclosure: None to report.

Informed consent was obtained for this case report.
